# Insight into m^6^A methylation from occurrence to functions

**DOI:** 10.1098/rsob.200091

**Published:** 2020-09-09

**Authors:** Wenxiu Ru, Xiaoyan Zhang, Binglin Yue, Ao Qi, Xuemei Shen, Yongzhen Huang, Xianyong Lan, Chuzhao Lei, Hong Chen

**Affiliations:** Key laboratory of Animal Genetics, Breeding and Reproduction of Shaanxi Province, College of Animal Science and Technology, Northwest A&F University, Yangling, Shaanxi 712100, People's Republic of China

**Keywords:** m^6^A methylation, writers, erasers, readers, functions

## Abstract

RNA m^6^A methylation is a post-transcriptional modification that occurs at the nitrogen-6 position of adenine. This dynamically reversible modification is installed, removed and recognized by methyltransferases, demethylases and readers, respectively. This modification has been found in most eukaryotic mRNA, tRNA, rRNA and other non-coding RNA. Recent studies have revealed important regulatory functions of the m^6^A including effects on gene expression regulation, organism development and cancer development. In this review, we summarize the discovery and features of m^6^A, and briefly introduce the mammalian m^6^A writers, erasers and readers. Finally, we discuss progress in identifying additional functions of m^6^A and the outstanding questions about the regulatory effect of this widespread modification.

## 1. Introduction

There has been extensive study of gene expression regulation. Chemical modification in DNA and RNA can regulate gene expression, which has evolved to ensure that the right genes are properly expressed for the conditions of a particular environment and at the necessary time. There has been awareness that the epigenetic modification of DNA can regulate gene expression and chromatin organization. This recently coined an additional regulatory layer termed ‘epitranscriptomics’ that depends on biochemical modifications to the RNA [1]. One of the most common RNA modifications is m^6^A methylation, or *N*^6^-methyladenosine, which refers to methylation of the adenosine base at the nitrogen-6 position. This methylation is a dynamically reversible modification that is installed, removed and recognized by methyltransferases, demethylases and readers, respectively [2]. This modification has been found in many eukaryotes, from plant to mammals, and even in viruses [3–6]. The m^6^A methylation is widely distributed in various RNA, with an average of three m^6^A sites per mRNA [7]. The m^6^A modification was first identified in the 1970s, but research on its potential function was initially limited owing to a lack of technologies for global detection of the m^6^A modification. In 2011, the obesity-associated protein (FTO) was found to effectively remove m^6^A modification on RNA [8], suggesting that m^6^A modification might serve a regulatory role. The development of next generation sequencing methods has facilitated further functional study of m^6^A modification.

**Figure 1.**
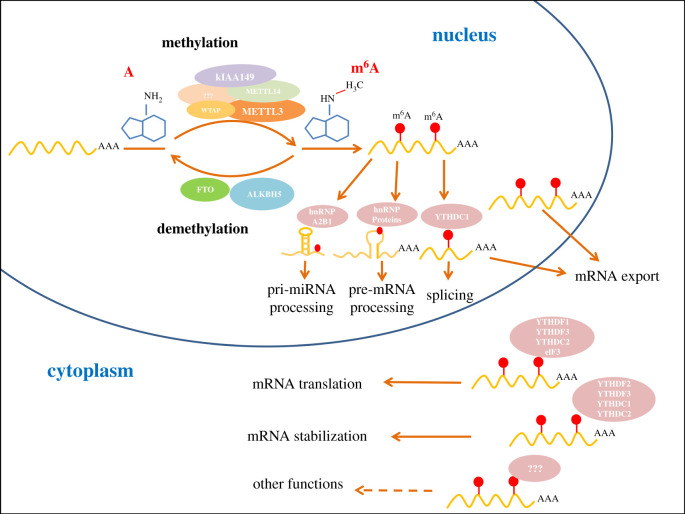
The patterns and functions of m^6^A methylation. The m^6^A methylation, occurs at the sixth N atom of RNA adenine, is installed by methyltransferase and erased by demethylase in the nucleus. The m^6^A readers that preferentially recognize m^6^A-containing RNA can impact the fate of the methylated RNA and give diverse regulatory function. In the nucleus, combination of m^6^A with hnRNP proteins or YTHDC1 can affect splicing of pre-mRNAs and combination with YTHDC1 mediates the export of methylated mRNA. In addition, combination with hnRNPA2B1 facilitates the processing of methylated pri-miRNA. In cytoplasm, YTHDF1, YTHDC2 and eIF3 bind to the methylated mRNAs to promote translation. YTHDF2, YTHDC1 and YTHDC2 bind to the methylated mRNAs to accelerate decay. Furthermore YTHDF3 combining with the YTHDF1 can promote targeted mRNA translation and combining with YTHDF2 can accelerate degradation. More m^6^A readers and other functions need to identify in m^6^A-modified mRNA.

In this review, we summarize the discovery and the main features of m^6^A modification, and briefly introduce the mammalian m^6^A writers, erasers and readers that interact with m^6^A sites to mediate the fate of mRNA (figure 1). We next describe the emerging knowledge of the functions of m^6^A in post-transcriptional gene expression regulation, animal development and cancer development. Finally, we discuss the emerging challenge and outstanding questions of this field, which should advance our understanding of m^6^A.

## 2. Discovery and features of m^6^A

In the 1970s, several groups characterizing mRNA 5ʹ structures in mammalian cells serendipitously discovered that polyadenylate RNA was rich in m^6^A modifications [9,10]. However, concerns about contamination from small amounts of known m^6^A sources, such as rRNA and small nucleolar RNAs [11–13], prevented confirmation of m^6^A as a ubiquitous modification in mRNA that is related to biogenesis [14]. In 2012, two groups of researchers firstly identified m^6^A peaks corresponding to 5678 mRNA transcripts and 6990 mRNA transcripts in mouse and human cells, and observed strong conservation of these m^6^A peaks in humans and mice [4,5]. The applied method was MeRIP-Seq or m^6^A-seq, which relies on the use of highly specific m^6^A antibodies to immunoprecipitate methylated mRNAs and then uses next generation sequencing to map methylated transcripts [4,5]. However this method lacks high sensitivity and resolution. The development of m^6^A-miCLIP and PA-m^6^A-seq methods have allowed more subtle mapping of m^6^A modification [15,16]. The technologies for detection and analysis of m^6^A sites continue to advance, providing more insight into the importance of this modification and its function in gene regulation.

Two mechanisms for regulating m^6^A deposition have been described to date. First, histone H3 trimethylation at Lys36 (H3K36me3) can globally regulate m^6^A deposition. Approximately 70% of m^6^A peaks are enriched near H3K36me3 sites. The depletion of H3K36me3 led to a reduction of the m^6^A level, because H3K36me3 is coupled with METTL14, which recruits the m^6^A methyltransferase complex to newly synthesized RNAs and with RNA polymerase II mediates the co-transcriptional deposition of m^6^A [17]. Second, transcription factors can mediate the dynamic level of m^6^A methylation. For example, Zfp217 can reduce the level of m^6^A by activating the demethylase FTO and SMAD2/D3 can recruit the m^6^A methyltransferase complex to newly synthesized RNA to facilitate m^6^A deposition [18,19].

There are several features of m^6^A modification: (i) mapping of m^6^A sites revealed that they preferentially map near stop codons, in the 3′ untranslated regions (UTRs), followed by the coding sequences (CDS) and the 5′ UTR regions [4,5]; (ii) the m^6^A motif was originally identified as (G/A) (m^6^A) C [20,21]. Recently, this motif has been more fully described as G [G/A] (m^6^A) CU, with almost 90% of the m^6^A peaks containing these motifs [4,5,22]; (iii) this modification is widely distributed among species including human, mammals, yeast, *Arabidopsis* and even viruses [3–6,23]; (iv) in addition to being found in mRNA, the m^6^A modification has been observed in tRNA, rRNA and other abundant non-coding RNA [24]. Signals for m^6^A have also been found in several classes of lncRNAs, including the well-known XIST and MALAT1 [4,5,25,26]. The m^6^A modification can also alter the expression of mature miRNA by affecting the production of pri-miRNA [27]. Recent studies have shown that intracellular m^6^A methylation can regulate the translation, destabilization, export and biogenesis of circRNAs [28–31]; and (v) in mammals, m^6^A modification is widely present in multiple tissues, with highest levels in liver, kidney and brain [5]. Recent work has shown that m^6^A and m^6^Am are highly specific to the brain, and some tissue-specific m^6^A signals may distinguish different human and mouse tissue types [32]. Overall, m^6^A modification is universal and exhibits organizational preference.

## 3. Cellular system of m^6^A methylation

### 3.1. m^6^A writers

The m^6^A modification is performed by a methyltransferase complex, or ‘writer’. This complex consists of two subunit complexes: an m^6^A-METTL complex (MAC) and an m^6^A-METTL-associated complex (MACOM), which can transfer the methyl group from *S*-adenosylmethionine (SAM) to the *N*^6^-amine of adenosine [33]. The m^6^A-METTL complex includes methyltransferase 3 (METTL3) and methyltransferase 14 (METTL14), which form a stable heterodimer. In 1997, a 70 kDa protein called MTA-70 (or METTL3) was successfully isolated and found to contain a classic SAM-binding methyltransferase domain (SAM) [34]. METTL3 is the catalytic subunit and binds to SAM [34] and METTL14 acts to stabilize the conformation and promote binding to RNA [35,36]. The lack of METTL3 could promote the apoptosis of HeLa cells and causes a decrease of m^6^A level [37]. METTL3 is highly conserved in eukaryotes and its homologues have been found in yeast, plants and flies [38–40]. Notably, the absence of METTL3 can block development in yeast and flies, and can lead to death in *Arabidopsis* and mice [39–41]. An early study revealed METTL14 is highly similar to METTL3 [42], and further research confirmed that METTL14 is also a methyltransferase [43]. METTL14 can synergistically increase METTL3 methyltransferase activities [43,44]. Interestingly, the knockdown of METTL14 resulted in a greater reduction in m^6^A levels than the knockdown of METTL3 in HeLa and 293T cells [43]. The METTL3/14 complex can selectively methylate RRACH sequences [43].

Subsequent efforts focused on m^6^A-METTL-associated complexes and how these complexes promote methyltransferase activities. The Wilms tumour-associated protein (WTAP) can interact with the METTL3/14 complex to promote mRNA methylation [43]. Although WTAP lacks methyltransferase activity *in vitro*, it promotes the localization of the METTL3/14 complex to nuclear speckles and facilitates mRNAs methylation [45]. Interfering with WTAP significantly reduces the level of m^6^A and prevents METTL3/14 complex localization to nuclear speckles [45]. KIAA1429 (also VIRMA) is a newly discovered component of the methyltransferase complex. Proteomic studies revealed important interactions with KIAA1429 and WTAP, and the absence of KIAA1429 substantially reduces the level of m^6^A modification [46,47]. Recent studies showed KIAA1429 is critical for the specific installation of m^6^A to 3′ UTR sites [48]. The RNA-binding protein 15/15B (RBM15/15B) preferentially binds to U-rich regions to recruit the m^6^A complex and may promote the methylation of specific RNA [25]. Another methyltransferase, METTL16, can install m^6^A on U6 snRNA and other highly structured ncRNAs and pre-mRNAs [49–51]. METTL16 may act a splicing enhancer to produce stable mature *MAT2A* mRNA encoding SAM synthetase during low-SAM conditions [49]. Recent study revealed the role of METTL16 in promoting early mouse embryonic development through regulation of SAM availability [52]. In *Arabidopsis*, HAKAI was identified as a new element by interaction with WTAP and was found necessary for m^6^A methylation [53,54]. The CCCH-type 13 zinc finger protein (ZC3H13) and its homologous protein FLacc in *Drosophila* are also involved in m^6^A installation by promoting the localization of WTAP and the deposition of m^6^A [55,56]. Most recently, ZCCHC4, a new m^6^A methyltransferase, was reported to methylate human 28S rRNA within the AAC motif [57]. The known m^6^A methyltransferases and their functions are listed in table 1.

**Table 1.** Functions of m^6^A writers and erasers.

	molecule	effect on m^6^A modification	other functions	references
m^6^A writers	METTL3	catalytic core of methyltransferase	enhances translation	[34,58,59]
	METTL14	stabilize METTL3/14 complex and promote the binding to RNA		[35,36]
	WTAP	promote the localization of METTL3/14 complex		[45]
	KIAA149(VIRMA)	interactions with WTAP and installation of m^6^A to the 3′ UTR		[48]
	RBM15/15B	binding to U-rich regions to recruit the methyltransferase complex		[25]
	METTL16	promote methylation of U6 snRNA, ncRNAs and pre-mRNAs	facilitate splicing of specific mRNA	[49–52]
	HAKAI	necessary for the m^6^A methylation in *Arabidopsis*		[53,54]
	ZC3H13	promote the WTAP localization and m^6^A deposition		[55,56]
	ZCCHC4	methylate human 28S rRNA		[57]
m^6^A erasers	FTO	remove m^6^A and m^6^Am	regulate pre-mRNA alternative splicing	[8,60,61]
	ALKBH5	remove m^6^A	regulate mRNA processing, metabolism and export	[62–64]

### 3.2. m^6^A erasers

Until endogenous enzymes capable of demethylation of m^6^A were found, m^6^A modification was regarded as a static modification. An important recent study identified FTO and ALKBH5 as m^6^A demethylases that can remove m^6^A methylation. These ‘erasers’ belong to the AlkB family and require the involvement of ferrous ion, α-ketoglutarate and oxygen [65,66]. FTO is associated with weight gain and obesity in humans [67]. Initial studies demonstrated that FTO could demethylate 3-methylthymidine (3mT) in single-stranded DNA and 3-methyluracil (3mU) in single-stranded RNA [65,68]. In 2011, FTO was shown to effectively remove m^6^A methylation of mRNA *in vitro* and inside cells [8]. Subsequent study revealed that FTO can produce two intermediates in removing m^6^A : N6-hydroxymethyladenosine (hm^6^A) and N6-formyladenosine (f^6^A), which is unrecognized by m^6^A ‘readers’ [69]. Knockdown of FTO in HeLa cells can increase the level of m^6^A and overexpression can reduce the level of m^6^A in mRNA [8]. More recently, FTO has been found to preferentially target intronic regions in pre-mRNAs rather than mRNAs, so can regulate pre-mRNA alternative splicing and 3′ UTR processing [60]. In addition to m^6^A, FTO can also effectively remove m^1^A from specific tRNAs and cap-m^6^Am from mRNAs and some snRNAs [61]. FTO has higher demethylation activity for m^6^Am and can stabilize the 5′ cap in mRNA, making an effect on mRNA stability likely [61]. Most recently, FTO was shown to remove m^6^Am methylation in snRNAs, suggesting that methylation information in snRNA may influence mRNA splicing [70].

Recently, ALKBH5 was identified as a second mammalian m^6^A demethylase [62]. ALKBH5 is enriched in the nucleus, unlike FTO, which is detected in the cytosol and nucleus [61,62]. Based on its localization, ALKBH5 may target nuclear RNAs and also interact with mRNA processing factors to regulate mRNA processing, metabolism and export [62,63]. The m^6^A demethylation process catalysed by ALKBH5 does not produce any intermediates. A lack of ALKBH5 in HeLa cells increased the m^6^A level by 9%, while overexpression of ALKBH5 decreased m^6^A level by 29% in total mRNA [62]. ALKBH5 was found to be highly expressed in the testicles of mice, and knockout of ALKBH5 inhibited spermatogenesis and decreased male fertility [62]. ALKBH5 can also modulate correct splicing and promote the production of longer 3′ UTR mRNAs in the nuclei of spermatocytes and round spermatids [64]. The known m^6^A demethylases and their functions are listed in table 1.

### 3.3. m^6^A readers

Although methyltransferase and demethylase endow the structural characteristics of RNA, m^6^A readers preferentially recognize m^6^A-containing mRNA, and impact the fate of target mRNA to give diverse regulatory functions. Recent studies have confirmed that m^6^A readers have a YTH domain that enables them to selectively target m^6^A-containing mRNA [71,72]. Proteins with a YTH domain for recognition of m^6^A-containing mRNA include: YTHDC1, YTHDC2, YTHDF1, YTHDF2 and YTHDF3. YTHDF2, which has the highest affinity to m^6^A, can selectively bind the m^6^A motif to regulate mRNA degradation [73]. Studies have found that mRNA bound to YTHDF2 can be transferred to an RNA degradation site using an N-terminal, such as the processor (p-body), and YTHDF2 can also directly recruit the CCR4-NOT deadenylase complex to accelerate degradation [73,74]. Several studies suggested that the IDR domain plays an effector function, where the IDR of YTHDF2 bound to mRNA allows targeting of P-bodies and also interaction with CCR4-NOT and endoribonuclease RNase P/MRP [29,73,74]. Importantly, YTHDF2 can block demethylation of 5′ UTR by FTO to stabilize methylation levels in cells [75]. A ratio of total mRNA by 21%, suggesting that the YTHDF2 destabilizes m^6^A-modified mRNA [73]. Related proteins YTHDF1 and YTHDF3 can promote translation by recruiting translation initiation factors in HeLa cells [76,77]. Knockout of YTHDF1 does not affect overall mRNA stability, but the overall translation efficiency is significantly reduced owing to interaction of YTHDF1 with eIF3 and other translation initiation factors [76]. Interestingly, YTHDF3 was proposed to complex with both YTHDF1 and YTHDF2 to promote mRNA translation and degradation upon binding its targets [78]. However, the mechanisms by which binding affects translation and degradation have not been fully described. YTHDC1, also known as YT521-B, has a variety of regulatory functions, including regulation of mRNA splicing [79], accelerating mRNA export [80], silencing the X chromosome [25] and promoting the decay of specific transcripts [81]. Recent studies have shown that YTHDC2 can increase the translation efficiency of its targets as well as decrease their mRNA abundance and is also involved in the regulation of meiosis and spermatogenesis [82,83].

In addition to the YTH domain family, eukaryotic initiation factor 3 (eIF3), a component of the 43S translation initiation complex, directly binds to the 5′ UTR of m^6^A mRNA and affects translation initiation [84]. Member of the heterogeneous nuclear ribonucleoprotein family, hnRNPC, hnRNPG, and hnRNPA2B1, were identified as m^6^A readers that regulate alternative splicing events [85–88]. The hnRNPC protein is a nuclear RNA-binding protein that is involved in the processing of pre-mRNA [89,90]. The m^6^A region of mRNA often lacks secondary structure which promotes hnRNPC binding to RNA, allowing it to regulate the abundance and alternative splicing of target genes [87,91]. Another member of the hnRNP family, hnRNPA2B1, was identified as an m^6^A binding protein that affects m^6^A-dependent alternative splicing and microRNA maturity [27,85]. The hnRNPG protein selectively binds m^6^A-modified RNA using Arg-Gly-Gly (RGG) motifs and interacts with RNA polymerase II (RNAPII) to regulate exon splicing [86,88]. In another class of m^6^A readers, insulin-like growth factor 2 binding protein 1-3 (IGF2BP1-3) and Prrc2a stabilize m^6^A-containing mRNA [92,93]. The known m^6^A readers and their functions are listed in table 2.

**Table 2.** Functions of m^6^A readers.

	molecule	functions	references
YTH domain family	YTHDF1	promote m^6^A-modified RNA translation	[77]
	YTHDF2	regulate m^6^A-modified RNA degradation	[29,73,74]
	YTHDF3	promote m^6^A-modified RNA translation and degradation	[77,78]
	YTHDC1	regulate m^6^A-modified RNA splicing, export and degradation	[79–81]
	YTHDC2	promote m^6^A-modified RNA translation and degradation	[82,83]
hnRNP family	hnRNPC	regulate the abundance and alternative splicing of target genes	[87]
	hnRNPG	regulate the alternative splicing of target genes	[86,88]
	hnRNPA2B1	regulate the alternative splicing of target genes and microRNA maturity	[27,85]
others	eIF3	promote m^6^A-modified RNA translation	[84]
	IGF2BP1–3	stabilize m^6^A-modified mRNA	[92]
	Prrc2a	stabilize m^6^A-modified mRNA	[93]

## 4. Biological function of m^6^A

With the improvement of m^6^A sequencing and detecting technology, many regulatory functions and mechanisms of m^6^A have been revealed in a variety of biological processes. Several studies have examined the biological function of m^6^A in gene expression regulation [94], organism development [95] and cancer development [96].

### 4.1. The regulation of gene expression

Modification by m^6^A regulates gene expression by affecting the splicing, translation, stability and localization of mRNA.

#### 4.1.1. mRNA splicing

The function of m^6^A was initially proposed to be the regulation of mRNA splicing because characterized m^6^A residues were observed in the nucleus and in introns of pre-mRNA, and because intron splicing can reduce the m^6^A level of total RNA [97,98]. Knockout of WTAP or METTL3 causes variable mRNA splicing isoforms [45]. Several m^6^A reader proteins can promote splicing events, including YTHDC1, which regulates splicing via recruiting other splicing-related proteins [79], as well as hnRNPC and hnRNPA2B1 that regulate splicing via binding to m^6^A-dependent structural switches [87,88]. Additionally, hnRNPG as splicing factors can interact with both nascent RNA and the carboxy-terminal domain (CTD) of RNAPII to regulate alternative splicing of m^6^A-modified RNA by hnRNPG binding and RNAPII occupancy [86]. In addition, ALKBH5 has been shown to affect splicing rates [62]. Further studies revealed that the deletion of METTL3 in mouse embryonic stem cells (mESCs) can reduce 0.5% of alternative splicing events [41,99]. Overall these results support a model in which m^6^A regulates mRNA splicing.

#### 4.1.2. mRNA translation

Early studies found significantly enrichment of ribosome-related components in m^6^A-containing mRNA that was not observed in mRNA without m^6^A [100]. The m^6^A reader YTHDF1 increases the translation efficiency of m^6^A-modified mRNA through direct interaction with translation initiation factors and ribosomal subunits [76,78]. Another YTH domain protein, YTHDF3, interacts with YTHDF1 in HeLa cells to promote translation, but a clear mechanism by which the combination of these two factors affect translation has not been described [78]. Notably, increased 5′ UTR methylation in the form of m^6^A can promote translation initiation independent of a 5′-end *N*^7^-methylguanosine cap [75]. Separately from catalytic activity, METTL3 enhances translation of bound RNA by directly recruiting translation initiation factors in an RNA-independent manner [58]. Most recently, a study showed that METTL3 can interact with the eIF3 h subunit at the 5′-end of mRNA bound to specific sites near the translation stop codon to facilitate circularization of the mRNA for ribosome recycling and translational control [59].

#### 4.1.3. mRNA stability

Knockdown of METTL3 and METTL14 led to a modest increase in stability of methylated transcripts, suggesting that m^6^A can influence mRNA stability [44]. Studies on the half-life of target mRNAs revealed a significant increase in stability when YTHDF2 was not present, indicating that YTHDF2 accelerates mRNA degradation. Importantly, YTHDF2 localized to P-bodies, a subset of cellular processing bodies [73]. Consistent with this view, a study found that YTHDF2 recruits CCR4-NOT through direct interaction with CNOT1 to promote degradation of methylated transcripts [74]. Another m^6^A reader involved in RNA degradation is YTHDC2. The researchers observed that a slight increase in the expression of m^6^A-modified transcripts in YTHDC2-knockout testes [83]. Unlike the m^6^A reader, m^6^A may regulate RNA stability by affecting its secondary structure. The RNA-binding protein HuR, which binds to the U-rich region of the 3′ UTR in mRNA, blocks binding of the miRNA and thus prevents degradation [101]. Studies have shown that m^6^A interferes with HuR binding in miRNA target genes, therefore promoting the degradation of mRNA. At the same time, knockout of METTL3 inhibits Ago2 binding to target mRNA and increases its stability [44]. A new mechanism of m^6^A-modified RNAs degradation was reported recently, in which HRSP12 acts as an adaptor to connect YTHDF2 and RNase P/MRP (endoribonucleases) resulting in endoribonucleolytic cleavage of YTHDF2-bound RNAs [29].

#### 4.1.4. mRNA export

Knockdown of METTL3 can prevent the nuclear export of circadian clock genes *Per2* and *Arntl,* resulting in a prolonged circadian period [102]. ALKBH5 is mainly localized in nuclear speckles and depletion of ALKBH5 can accelerate the nuclear export of target RNAs [62]. Combined depletion of WTAP and KIAA1429 led to a nuclear accumulation of specific m^6^A-modified transcripts [103]. The m^6^A reader YTHDC1 mediates the export of methylated mRNA from the nucleus to the cytoplasm in human cells. YTHDC1 can interact with SRSF3 and SRSF3 interacts with the nuclear export receptor NXF1 to mediate the export of mRNA. The knockdown of YTHDC1 does not affect the overall level of m^6^A, but does result in nuclear accumulation of mRNA [80]. The nuclear export of mRNA is controlled by the TREX complex and the heterodimeric nuclear export receptor NXF1-P15 [104]. The m^6^A writer complex can recruit TREX to m^6^A-modified mRNAs and TREX can stimulate recruitment of YTHDC1 and NXF1, resulting in the export of mRNA [103]. Both Zika virus and HIV-1 have a high level of m^6^A methylation, with accelerated nuclear export, and other processing steps dependent on m^6^A during replication, suggesting that methylation may be necessary for nuclear export of mRNA [105].

### 4.2. Organism development

Growing research indicates that m^6^A modification is necessary for early embryo development. Early studies showed a lack of Ime4 in *Drosophila*, the METTL3 homologous protein, has a semi-lethal effect on development, and the fertility of adult individuals is reduced owing to impaired NOTCH signalling [40]. Recent study indicates that depletion of Ime4 in *Drosophila* does not really cause prominent lethality in adults. The study showed that m^6^A methyltransferase plays a critical regulator in controlling neuronal functions and sex determination by its nuclear reader YT521-B [106]. However, depletion of METTL3 in mice has a lethal effect on embryonic development [41]. Furthermore, in *Arabidopsis*, the absence of the orthologue of the yeast and human mRNA adenosine methylase (MTA) can affect embryonic development and in yeast, Ime4 plays an important role in cell meiosis [23,107]. During the maternal to zygomatic transition (MZT) in zebrafish, maternal mRNAs with m^6^A modification were rapidly cleared by YTHDF2. Knockout of YTHDF2 increased the stability of maternal mRNAs and prevented the egg transforming into the fertilized state, ultimately slowing the embryo from entering the MZT and delaying development of the offspring zebrafish [108]. These studies indicate that m^6^A modification is required for early embryo development in animals.

Recent studies have shown that m^6^A is involved in various physiological processes, such as stem cell self-renewal and differentiation, lipid metabolism, glucose metabolism, DNA damage repair, control of heat shock response, and circadian rhythm. The lack of YTHDF1 can impair hippocampal-dependent neurological functions in mice such as spatial learning and memory, but overexpression of YTHDF1 in the hippocampus can restore this damage. It was showed that binding of YTHDF1 to methylated transcripts can promote the function of synaptic transmission and long-term potentiation genes [109]. After an organism was subjected to heat shock, METTL3 rapidly bound to heat shock genes and YTHDF2 can compete with the FTO to prevent 5′ UTR demethylation, thus enhancing translation [75]. Similarly, after DNA ultraviolet damage, transcripts methylated by METTL3 are rapidly localized at the site of injury, and then recruit DNA polymerase κ (Pol κ) to promote damage repair [110]. The process of m^6^A methylation also plays a vital physiological role in the circadian rhythm cycle. Reduced m^6^A can prevent the nuclear export of circadian clock genes *Per2* and *Arntl* [102]. There are many reported roles of m^6^A in lipid metabolism. FTO-dependent demethylation led to lipid accumulation and triglyceride deposition in skeletal muscle cells and hepatocytes [111,112]. FTO can also affect glucose metabolism by reducing the m^6^A level of *FOXO1*, an important transcription factor that regulates hepatic gluconeogenesis [113].

Many studies have emphasized a role of m^6^A in the regulation of stem cell differentiation. Earlier research showed that knockdown of METTL3 or METTL14 reduced the level of m^6^A and self-renewal in mESCs [44]. However, conflicting results indicated that knockout of METTL3 in mESCs increased self-renewal and impaired differentiation towards cardiomyocytes and neurons by enhancing the level of regulator *Nanog* necessary for self-renewal [114]. The knockout of METTL3 in early mouse embryos failed to transform naive mESCs into the primed state, resulting in post-implantation embryo death. However the knockdown of METTL3 at a primed pluripotency state promoted differentiation [41]. This result suggests that m^6^A may serve as a switch to regulate the expression of multiple pluripotency genes and developmental regulators in early embryos. Similarly in mouse embryonic fibroblasts, knockdown of METTL3 resulted in a decrease in m^6^A abundance and improved reprogramming efficiency [115]. In addition, in haematopoietic stem cells (HSCs) the knockout of YTHDF2 can maintain the function of HSCs by regulating the stability of multiple mRNAs critical for HSC self-renewal [116]. Taken together, these results suggested that m^6^A is required for maintaining pluripotency and stem cell differentiation.

### 4.3. Cancer development

The process of m^6^A methylation has been related to the development of human diseases, especially cancer proliferation, apoptosis and metastasis. The deletion of WTAP in a human acute myeloid leukaemia (AML) cell line reduced proliferation and increased differentiation and apoptosis [117]. Consistent with that effect of WTAP deletion, deletion of METTL3 in the AML cell line promoted cell differentiation and apoptosis by reducing translation of METTL3-binding genes, including *MYC*, *BCL2* and *PTEN* [118]. Meanwhile, METTL14 play an oncogenic role in the AML cell line by regulating m^6^A methylation of tumour genes *MYB* and *MYC* [119]. However, the reduction of m^6^A plays an oncogenic role in some AMLs. In the t (11q23), t (15; 17), and FLT3-ITD type AML cell lines, FTO is highly expressed, which can promote leukemogenesis. FTO can also suppress AML cell differentiation induced by all-trans-retinoic acid (ATRA) treatment [120].

In breast cancer (BC), HBXIP can upregulate METTL3 by suppressing miRNA let-7 g, and METTL3 promotes HBXIP expression through increased m^6^A modification, leading to an accelerated proliferation of BC cells [121]. In human pancreatic cancer (PC), YTHDF2 is upregulated to promote cancer cell proliferation and the epithelial-mesenchymal transition (EMT) [122]. The overall m^6^A level was significantly enriched in PC cells and overexpression of ALKBH5 can inhibit cancer cell migration and invasion by demethylating lncRNA KCNK15-AS1 [123]. In human hepatoma cells (HCC), decreased m^6^A and METTL14 was detected and overexpression of METTL14 can interact with DGCR8 to regulate the maturation of pri-miRNA126 in an m^6^A-dependent manner, reducing the metastasis of hepatoma cells [124]. However, METTL3 is upregulated in HCC and knockdown of METTL3 significantly inhibits the proliferation, migration and metastasis of cancer cells. The results indicated that METTL3 can increase m^6^A abundance in *SOCS2* mRNA, with degradation that is dependent on YTHDF2, which ultimately promoted liver cancer [125]. The conflicting results demonstrate that more work remains to explore the function of m^6^A methylation.

Glioblastoma stem cells (GSCs) possess self-renewal and differentiation capabilities in malignant tumours. The knockdown of METTL3 and METTL14 in GSCs reduced the m^6^A level, thereby enhancing the expression of oncogenes including *ADAM19*, *EPHA3* and *KLF4*, which promoted cell growth and self-renewal. However, overexpression of METTL3 or knockdown of FTO inhibited GSC growth and self-renewal, resulting in inhibition of tumorigenesis [126]. The latest research has shown that m^6^A modification affects tumour antigen-specific T cell immune responses by regulating the translation efficiency of lysosomal cathepsin in dendritic cells [127]. Knockout of YTHDF1 in mice enhances the response of tumour antigen-specific CD8+ T cells. Further study showed that the mRNAs of multiple lysosomal cathepsins are m^6^A modified and methylated transcripts can be recognized by YTHDF1, resulting in increased translation [127].

Recent studies provide evidence that m^6^A methylation may be used as a potential prognostic biomarker of the tumour. In gastric cancer, reduced m^6^A modification can promote gastric cancer malignancy by activating oncogenic signalling, and FTO acting as an oncogene can promote tumour growth [128,129]. Similarly, hnRNPC was identified as an independent prognostic biomarker in oral squamous cell carcinoma (OSCC) and the overexpression of hnRNPC facilitated the development of OSCC cells *in vitro* [130]. MALDI-TOF-MS revealed that the methylation level of miRNA methylation was significantly higher in PC compared with normal tissues, so evaluating miRNA methylation is a promising diagnostic strategy [131]. However, whether m^6^A methylation can serve as a molecular tool to regulate gene expression for treatment of human diseases is a key question to be addressed.

## 5. Conclusion

Similar to DNA methylation, RNA m^6^A methylation is a dynamic reversible modification catalysed by methyltransferase and demethylase, where the proteins that recognize this modification alter the function of the target mRNA. With improved technology to detect and analyse m^6^A, recent years have witnessed a rapid advance in studies on m^6^A methylation. The m^6^A methylation is widely found in various RNAs in both prokaryotes and eukaryotes, and m^6^A methylation can regulate RNA stabilization, transport, splicing and translation. In addition, m^6^A methylation can alter RNA structures to affect the interaction of mRNA binding proteins [88]. Additionally, m^6^A is closely related to embryonic development, cancer metastasis, immune response, stem cell self-renewal differentiation, lipid metabolism, glucose metabolism, DNA damage repair, heat shock response control and circadian rhythm control.

Several challenging questions about m^6^A methylation remain to be addressed. In mammals a consensus sequence of m^6^A: G [G/A] (m^6^A) CU has been defined. However, although this consensus motif is ubiquitous in the transcriptome, only a fraction of these sites are methylated *in vivo*. Thus, we still need to elucidate the mechanisms for selective specificity in the m^6^A-modified transcripts. The result of this selection may be related to different requirements for development and environmental stimuli. Functions of m^6^A may vary in different environmental stimuli or cellular type. For example, in heat stress, the level of m^6^A increases in the 5′ UTR and promotes the expression of *HSF* by initiating independent-cap translation, thus promoting the response of the cell heat shock pathway [75]. This dynamical change probably leads to different fates of the methylated RNAs for various environmental stimuli or cellular types. Additionally, the diversity in the binding classes of m^6^A readers also can change the fate of methylated transcripts. YTHDF3 has two different functions, acting to promote targeted mRNA translation with YTHDF1 and acting to accelerate degradation with YTHDF2 [78]. The mechanism by which YTHDF3 combines with YTHDF1 or YTHDF2 remains unclear. This complexity means that simply exploring the m^6^A functions in single environmental systems may not adequately reveal the comprehensive roles of m^6^A methylation in multiple biological processes. YTHDC1 possesses a variety of regulatory functions, but how YTHDC1 selects different sets of m^6^A-modified RNA is unclear [79–81]. Indeed, there is limited understanding of how m^6^A readers identify and select their target transcripts to modulate the fate of modified RNAs. In addition, METTL3 and ALKBH5 have regulatory functions for modified RNAs that are independent of both catalytic activity and m^6^A readers [62,59]. Thus, we should continue to investigate the function and molecular mechanisms of m^6^A methylase to better understand this complex process. Collectively, m^6^A methylation needs us to uncover the occurrence and function from different layers.

## Data accessibility

This article has no additional data.
